# Supplementary material for: Hybrid Models and Biological Model Reduction with PyDSTool
Source: PLoS Comput Biol. 2012 Aug 9;8(8):e1002628. doi: 10.1371/journal.pcbi.1002628 (PMC3415397; doi:10.1371/journal.pcbi.1002628)
Supplement: Text S4 — Complete source code for the PyDSTool package (version 0.88.120504). Includes API documentation and help files linking to web pages. This file is identical to the current public release on Sourceforge.net. (ZIP) [file pcbi.1002628.s004.zip › PyDSTool/html/PyDSTool.Generator.Radau_ODEsystem'-pysrc.html]

xml version="1.0" encoding="ascii"?


PyDSTool.Generator.Radau\_ODEsystem'


| Home | Trees | Indices | Help | PyDSTool |
| --- | --- | --- | --- | --- |

Package PyDSTool ::
Package Generator ::
Module Radau\_ODEsystem'

[hide private]

[frames] | no frames]

# Source Code for Module PyDSTool.Generator.Radau\_ODEsystem'

```
   1  # Radau ODE system
 
   2  from __future__ import division 
   3  
 
   4  from allimports import * 
   5  from PyDSTool.Generator import ODEsystem as ODEsystem 
   6  from baseclasses import Generator, theGenSpecHelper, genDB, _pollInputs 
   7  from PyDSTool.utils import * 
   8  from PyDSTool.common import * 
   9  from PyDSTool.ModelSpec import QuantSpec 
  10  from PyDSTool.integrator import integrator 
  11  from PyDSTool.parseUtils import addArgToCalls, wrapArgInCall 
  12  import PyDSTool.Redirector as redirc 
  13  import numpy as npy 
  14  
 
  15  # Other imports
 
  16  from numpy import Inf, NaN, isfinite, int, int32, float, float64, \
 
  17      sometrue, alltrue, any, all, concatenate, transpose, array, zeros 
  18  import math, random 
  19  import operator 
  20  from copy import copy, deepcopy 
  21  import os, platform, shutil, sys, gc 
  22  # Use numpy.distutils that supports fortran compiler
 
  23  #import numpy.distutils
 
  24  from numpy.distutils.core import setup, Extension 
  25  from distutils.sysconfig import get_python_inc 
  26  
 
  27  # path to the installation
 
  28  import PyDSTool 
  29  _pydstool_path = PyDSTool.__path__[0] 
  30  
 
  31  
 
  32  rout = redirc.Redirector(redirc.STDOUT) 
  33  rerr = redirc.Redirector(redirc.STDERR) 
  34  
 


35 -def distutil_destination():


36      """Makes the goofy destination directory string so that we can find where the distutils
 
  37      fortran compiler puts things.""" 
  38      osname = str.lower(platform.system()) 
  39      pyname = platform.python_version_tuple() 
  40      machinename = platform.machine() 
  41      if osname == 'linux': 
  42          destdir = 'src.'+osname+'-'+machinename+'-'+pyname[0] + '.' + pyname[1] 
  43      elif osname == 'darwin': 
  44          osver = platform.mac_ver()[0].split('.') 
  45          if int(scipy.__version__.split('.')[1]) > 5 and len(osver)>1 and osver != ['']: 
  46              destdir = 'src.macosx-'+osver[0]+'.'+osver[1]+'-'+machinename+'-'+pyname[0] + '.' + pyname[1] 
  47          else: 
  48              destdir = 'src.'+osname+'-'+platform.release()+'-'+machinename+'-'+pyname[0] + '.' + pyname[1] 
  49      elif osname == 'windows': 
  50          destdir = 'src.win32-'+pyname[0]+'.'+pyname[1] 
  51      else: 
  52          destdir = '' 
  53  
 
  54      return destdir

  55  
 
  56  
 


57 -class radau(integrator):


58      """Radau 5 specialization of the basic integrator class.""" 
  59  
 


60 -    def __init__(self, modname, rhs='default_name', phaseDim=0, paramDim=0,
 
  61                   nAux=0, nEvents=0, nExtInputs=0, hasJac=0, hasJacP=0,
 
  62                   hasMass=0, extraSpace=0, defaultBound=1e8):


63          integrator.__init__(self, rhs=rhs, phaseDim=phaseDim, paramDim=paramDim,
 
  64                              nAux=nAux, nEvents=nEvents, nExtInputs=nExtInputs, hasJac=hasJac,
 
  65                              hasJacP=hasJacP, hasMass=hasMass, extraSpace=extraSpace,
 
  66                              defaultBound=defaultBound) 
  67          self.modname = modname 
  68          try: 
  69              self._integMod = __import__(modname, globals()) 
  70          except: 
  71              print "Error in importing compiled vector field and integrator." 
  72              print "Did you compile the RHS C code?" 
  73              raise 
  74          # check module's directory
 
  75          assert 'Integrate' in dir(self._integMod), \
 
  76                 "radau library does not contain Integrate()" 
  77  
 
  78          self.safety = [] 
  79          self.jacRecompute = [] 
  80          self.newtonStop = [] 
  81          self.stepChangeLB = [] 
  82          self.stepChangeUB = [] 
  83          self.stepSizeLB = [] 
  84          self.stepSizeUB = [] 
  85          self.hessenberg = [] 
  86          self.maxNewton = [] 
  87          self.newtonStart = [] 
  88          self.index1dim = [] 
  89          self.index2dim = [] 
  90          self.index3dim = [] 
  91          self.stepSizeStrategy = [] 
  92          self.DAEstructureM1 = [] 
  93          self.DAEstructureM2 = [] 
  94  
 
  95          retval = self._integMod.InitBasic(self.phaseDim, self.paramDim, self.nAux,
 
  96                                            self.nEvents, self.nExtInputs, self.hasJac,
 
  97                                            self.hasJacP, self.hasMass, self.extraSpace) 
  98  
 
  99          if retval[0] != 1: 
 100              raise PyDSTool_InitError('Call to InitBasic failed! (radau)') 
 101  
 
 102          self.initBasic = True

 103  
 
 104  
 


105 -    def Run(self, hinit=0, hmax=1.0, checkAux=0, calcSpecTimes=0, verbose=0,
 
 106              safety=0.9, jacRecompute=0.001, newtonStop=-1, stepChangeLB=1,
 
 107              stepChangeUB=1.2, stepSizeLB=0.2, stepSizeUB=8.0, hessenberg=0,
 
 108              maxNewton=7, newtonStart=0, index1dim=-1, index2dim=0, index3dim=0,
 
 109              stepSizeStrategy=1, DAEstructureM1=0, DAEstructureM2=0, useJac=0, useMass=0):


110          if not self.initBasic: 
 111              raise PyDSTool_InitError('initBasic is False (radau)') 
 112          if not self.initEvents: 
 113              raise PyDSTool_InitError('initEvents is False (radau)') 
 114          if not self.initIntegrate: 
 115              raise PyDSTool_InitError('initInteg is False (radau)') 
 116          if not self.setParams: 
 117              raise PyDSTool_InitError('setParams is False (radau)') 
 118          if self.nExtInputs > 0 and not self.initExtInputs: 
 119              raise PyDSTool_InitError('initExtInputs is False (radau)') 
 120  
 
 121          self.setRadauParams(hinit=hinit, hmax=hmax, checkAux=checkAux,
 
 122                              calcSpecTimes=calcSpecTimes,
 
 123                              verbose=verbose, safety=safety,
 
 124                              jacRecompute=jacRecompute, newtonStop=newtonStop,
 
 125                              stepChangeLB=stepChangeLB, stepChangeUB=stepChangeUB,
 
 126                              stepSizeLB=stepSizeLB, stepSizeUB=stepSizeUB,
 
 127                              hessenberg=hessenberg,maxNewton=maxNewton,
 
 128                              newtonStart=newtonStart, index1dim=index1dim,
 
 129                              index2dim=index2dim, index3dim=index3dim,
 
 130                              stepSizeStrategy=stepSizeStrategy,
 
 131                              DAEstructureM1=DAEstructureM1,
 
 132                              DAEstructureM2=DAEstructureM2,
 
 133                              useJac=useJac,useMass=useMass) 
 134  
 
 135          # For a run, we want to ensure indices are set to 0
 
 136          self.Reset() 
 137          T, P, A, Stats, H, Err, EvtT, EvtP = self._integMod.Integrate(self.ic,
 
 138                                                        self.t0,
 
 139                                                        self.hinit,
 
 140                                                        self.hmax,
 
 141                                                        self.safety,
 
 142                                                        self.jacRecompute,
 
 143                                                        self.newtonStop,
 
 144                                                        self.stepChangeLB,
 
 145                                                        self.stepChangeUB,
 
 146                                                        self.stepSizeLB,
 
 147                                                        self.stepSizeUB,
 
 148                                                        self.hessenberg,
 
 149                                                        self.maxNewton,
 
 150                                                        self.newtonStart,
 
 151                                                        self.index1dim,
 
 152                                                        self.index2dim,
 
 153                                                        self.index3dim,
 
 154                                                        self.stepSizeStrategy,
 
 155                                                        self.DAEstructureM1,
 
 156                                                        self.DAEstructureM2,
 
 157                                                        self.useJac,
 
 158                                                        self.useMass,
 
 159                                                        self.verbose,
 
 160                                                        self.checkAux,
 
 161                                                        self.calcSpecTimes) 
 162          self.points = P 
 163          self.times = T 
 164          self.auxPoints = A 
 165          self.eventTimes = EvtT 
 166          self.eventPoints = EvtP 
 167          self.errors = Err 
 168          self.stats = Stats 
 169          self.step = H 
 170  
 
 171          try: 
 172              self.lastTime = self.times[-1] 
 173              self.lastPoint = [self.points[i][-1] for i in range(self.phaseDim)] 
 174              self.lastStep = self.step 
 175          except IndexError: 
 176              self.lastTime = self.t0 
 177              self.lastPoint = self.ic 
 178              self.lastStep = self.hinit 
 179          self.numRuns += 1 
 180          self.canContinue = True 
 181  
 
 182          return T, P, A, Stats, H, Err, EvtT, EvtP

 183  
 
 184  
 


185 -    def Continue(self, tend, params=[], calcSpecTimes=0, verbose=0,
 
 186                   extInputChanged=False, extInputVals=[], extInputTimes=[],
 
 187                   bounds=[]):


188          if not self.initBasic: 
 189              raise PyDSTool_InitError('initBasic is False (radau)') 
 190          if not self.initEvents: 
 191              raise PyDSTool_InitError('initEvents is False (radau)') 
 192          if not self.initIntegrate: 
 193              raise PyDSTool_InitError('initInteg is False (radau)') 
 194          if not self.setParams: 
 195              raise PyDSTool_InitError('setParams is False (radau)') 
 196          if self.nExtInputs > 0 and not self.initExtInputs: 
 197              raise PyDSTool_InitError('initExtInputs is False (radau)') 
 198  
 
 199          if not self.canContinue: 
 200              raise PyDSTool_ContError('Unable to continue trajectory -- '
 
 201                          'have you run the integrator and reset events, etc?') 
 202  
 
 203          self.setContParams(tend=tend, params=copy(params),
 
 204                             calcSpecTimes=calcSpecTimes, verbose=verbose, extInputChanged=extInputChanged,
 
 205                             extInputVals=copy(extInputVals), extInputTimes=copy(extInputTimes),
 
 206                             bounds=copy(bounds)) 
 207  
 
 208          # For a continue, we do not set indices to 0
 
 209          T, P, A, Stats, H, Err, EvtT, EvtP = \
 
 210                  self._integMod.Integrate(self.lastPoint,
 
 211                                        self.lastTime,
 
 212                                        self.lastStep, self.hmax,
 
 213                                        self.safety,
 
 214                                        self.jacRecompute,
 
 215                                        self.newtonStop,
 
 216                                        self.stepChangeLB,
 
 217                                        self.stepChangeUB,
 
 218                                        self.stepSizeLB,
 
 219                                        self.stepSizeUB,
 
 220                                        self.hessenberg,
 
 221                                        self.maxNewton,
 
 222                                        self.newtonStart,
 
 223                                        self.index1dim,
 
 224                                        self.index2dim,
 
 225                                        self.index3dim,
 
 226                                        self.stepSizeStrategy,
 
 227                                        self.DAEstructureM1,
 
 228                                        self.DAEstructureM2,
 
 229                                        self.useJac,
 
 230                                        self.useMass,
 
 231                                        self.verbose,
 
 232                                        self.checkAux,
 
 233                                        self.calcSpecTimes) 
 234  
 
 235          self.points = P 
 236          self.times = T 
 237          self.auxPoints = A 
 238          self.eventTimes = EvtT 
 239          self.eventPoints = EvtP 
 240          self.errors = Err 
 241          self.stats = Stats 
 242          self.step = H 
 243  
 
 244          try: 
 245              self.lastTime = self.times[-1] 
 246              self.lastPoint = [self.points[i][-1] for i in range(self.phaseDim)] 
 247              self.lastStep = self.step 
 248          except IndexError: 
 249              self.lastTime = self.t0 
 250              self.lastPoint = self.ic 
 251              self.lastStep = self.hinit 
 252          self.numRuns += 1 
 253          self.numContinues += 1 
 254          self.canContinue = True 
 255  
 
 256          return T, P, A, Stats, H, Err, EvtT, EvtP

 257  
 
 258  
 


259 -    def setRadauParams(self, hinit, hmax, checkAux, calcSpecTimes,
 
 260                         verbose, safety, jacRecompute, newtonStop,
 
 261                         stepChangeLB, stepChangeUB, stepSizeLB, stepSizeUB,
 
 262                         hessenberg, maxNewton, newtonStart, index1dim,
 
 263                         index2dim, index3dim, stepSizeStrategy,
 
 264                         DAEstructureM1, DAEstructureM2, useJac, useMass):


265          useJac = int(useJac) 
 266          useMass = int(useMass) 
 267          checkAux = int(checkAux) 
 268          calcSpecTimes = int(calcSpecTimes) 
 269          hessenberg = int(hessenberg) 
 270  
 
 271          if not isinstance(hinit, _num_types): 
 272              raise TypeError("hinit must be int, float") 
 273  
 
 274          if not isinstance(hmax, _num_types): 
 275              raise TypeError("hmax must be int, float") 
 276  
 
 277          if abs(hinit) > abs(hmax): 
 278              raise ValueError("Abs value of hinit (%g) must be less than hmax (%g)"%(hinit,hmax)) 
 279  
 
 280          if not isinstance(checkAux, _int_types): 
 281              raise TypeError("checkAux must be int") 
 282          if checkAux not in (0,1): 
 283              raise TypeError("checkAux must be 0 or 1") 
 284          if checkAux == 1 and self.nAux <= 0: 
 285              raise ValueError("checkAux cannot be 1 if nAux is 0") 
 286  
 
 287          if not isinstance(verbose, _int_types): 
 288              raise TypeError("verbose must be int") 
 289          if verbose not in (0,1): 
 290              if verbose >= 2: 
 291                  # interpret all greater values as 1
 
 292                  verbose = 1 
 293              else: 
 294                  raise TypeError("verbose must be 0 or 1") 
 295  
 
 296          if not isinstance(calcSpecTimes, _int_types): 
 297              raise TypeError("calcSpecTimes must be int") 
 298          if calcSpecTimes not in (0,1): 
 299              raise TypeError("calcSpecTimes must be 0 or 1") 
 300          if calcSpecTimes == 1 and len(self.specTimes) <= 0: 
 301              raise ValueError("calcSpecTimes cannot be 1 if specTimes is empty") 
 302  
 
 303          if safety < 0: 
 304              raise ValueError("safety must be non-negative") 
 305          if jacRecompute <= 0.0: 
 306              raise ValueError("jacRecompute must be positive") 
 307          if newtonStop < 0: 
 308              newtonStop = 0 
 309          if stepChangeLB <= 0: 
 310              raise ValueError("stepChangeLB must be positive") 
 311          if stepChangeUB <= 0: 
 312              raise ValueError("stepChangeUB must be positive") 
 313          if stepSizeLB <= 0: 
 314              raise ValueError("stepSizeLB must be positive") 
 315          if stepSizeUB <= 0: 
 316              raise ValueError("stepSizeUB must be positive") 
 317  
 
 318          if stepChangeLB > stepChangeUB:   # was >= but this allows fac1=fac2=1 
 319              raise ValueError("stepChangeLB must be less than stepChangeUB") 
 320          if stepSizeLB >= stepSizeUB: 
 321              raise ValueError("stepSizeLB must be less than stepSizeUB") 
 322  
 
 323          if hessenberg not in (0,1): 
 324              raise ValueError("hessenberg must be 0 or 1") 
 325          if hessenberg == 1 and useMass != 0: 
 326              raise ValueError("hessenberg form cannot be used for implicit systems (mass matrix)") 
 327          if not isinstance(maxNewton, _int_types): 
 328              raise TypeError("maxNewton must be int") 
 329          if maxNewton <= 0: 
 330              raise ValueError("maxNewton must be positive") 
 331  
 
 332          if newtonStart not in (0,1): 
 333              raise ValueError("newtonStart must be 0 or 1") 
 334  
 
 335          if index1dim <= 0: 
 336              index1dim = self.phaseDim 
 337          if index2dim != 0: 
 338              raise ValueError("Currently index2dim must be 0") 
 339          if index3dim != 0: 
 340              raise ValueError("Currently index3dim must be 0") 
 341  
 
 342          if stepSizeStrategy not in (1,2): 
 343              raise ValueError("stepSizeStrategy must be 1 or 2") 
 344  
 
 345          if DAEstructureM1 != 0: 
 346              raise ValueError("Currently DAEstructureM1 must be 0") 
 347          if DAEstructureM2 != 0: 
 348              raise ValueError("Currently DAEstructureM2 must be 0") 
 349  
 
 350          if useJac not in (0,1): 
 351              raise ValueError("useJac must be 0 or 1") 
 352          if useMass not in (0,1): 
 353              raise ValueError("useMass must be 0 or 1") 
 354  
 
 355          if useJac == 1 and self.hasJac != 1: 
 356              raise ValueError("useJac must be 0 if hasJac is not 1") 
 357          if useMass == 1 and self.hasMass != 1: 
 358              raise ValueError("useMass must be 0 if hasMass is not 1") 
 359  
 
 360          self.hinit = hinit 
 361          self.hmax = hmax 
 362          self.safety = safety 
 363          self.jacRecompute = jacRecompute 
 364          self.newtonStop = newtonStop 
 365          self.stepChangeLB = stepChangeLB 
 366          self.stepChangeUB = stepChangeUB 
 367          self.stepSizeLB = stepSizeLB 
 368          self.stepSizeUB = stepSizeUB 
 369          self.hessenberg = hessenberg 
 370          self.maxNewton = maxNewton 
 371          self.newtonStart = newtonStart 
 372          self.index1dim = index1dim 
 373          self.index2dim = index2dim 
 374          self.index3dim = index3dim 
 375          self.stepSizeStrategy = stepSizeStrategy 
 376          self.DAEstructureM1 = DAEstructureM1 
 377          self.DAEstructureM2 = DAEstructureM2 
 378          self.useJac = useJac 
 379          self.useMass = useMass 
 380          self.verbose = verbose 
 381          self.checkAux = checkAux 
 382          self.calcSpecTimes = calcSpecTimes

 383  
 
 384  
 
 385  
 


386 -class Radau_ODEsystem(ODEsystem):


387      """Wrapper for Radau integrator (with support for differential-algebraic equations).
 
 388  
 
 389      Uses C target language only for functional specifications""" 
 390      _paraminfo = {'rtol': 'Relative error tolerance.',
 
 391                    'atol': 'Absolute error tolerance.',
 
 392                    'safety': 'Safety factor in the step size prediction, default 0.9.',
 
 393                    'max_step': 'Maximal step size, default tend-tstart.',
 
 394                    'init_step': 'Initial step size, default is a guess computed by the function init_step.',
 
 395                    'fac1': 'Parameter for step size selection; the new step size is chosen subject to the restriction  fac1 <= new_step/old_step <= fac2. Default value is 1.0.',
 
 396                    'fac2': 'Parameter for step size selection; the new step size is chosen subject to the restriction  fac1 <= new_step/old_step <= fac2. Default value is 1.2.',
 
 397                    'stepLB': '',
 
 398                    'stepUB': '',
 
 399                    'refine': 'Refine output by adding points interpolated using the RK4 polynomial (0, 1 or 2).',
 
 400                    'step_strategy': """Switch for step size strategy;
 
 401  If step_strategy=1  mod. predictive controller (Gustafsson).
 
 402  If step_strategy=2  classical step size control.
 
 403  The default value (for step_strategy=0) is step_strategy=1.
 
 404  the choice step_strategy=1 seems to produce safer results;
 
 405  for simple problems, the choice step_strategy=2 produces
 
 406  often slightly faster runs.""",
 
 407                    'jac_recompute': """Decides whether the Jacobian should be recomputed;
 
 408  increase jac_recompute to 0.1 say, when Jacobian evaluations
 
 409  are costly. for small systems jac_recompute should be smaller
 
 410  (0.001, say). negative jac_recompute forces the code to
 
 411  compute the Jacobian after every accepted step.
 
 412  Default 0.001.""",
 
 413                    'newton_start': "",
 
 414                    'newton_stop': "",
 
 415                    'max_newton': "Maximum number of Newton iterations to take in solving the implicit system at each step (default 7)",
 
 416                    'DAEstructureM1': "",
 
 417                    'DAEstructureM2': "",
 
 418                    'hessenberg': "",
 
 419                    'index1dim': "",
 
 420                    'index2dim': "",
 
 421                    'index3dim': "",
 
 422                    'use_special': "Switch for using special times",
 
 423                    'specialtimes': "List of special times to use during integration",
 
 424                    'check_aux': "Switch",
 
 425                    'extraspace': ""
 
 426                    } 
 427  
 


428 -    def __init__(self, kw):


429          """Use the nobuild key to postpone building of the library, e.g. in
 
 430          order to provide additional build options to makeLibSource and
 
 431          compileLib methods or to make changes to the C code by hand.
 
 432          No build options can be specified otherwise.""" 
 433  
 
 434          if 'nobuild' in kw: 
 435              nobuild = kw['nobuild'] 
 436              # delete because not covered in ODEsystem
 
 437              del kw['nobuild'] 
 438          else: 
 439              nobuild = False 
 440          ODEsystem.__init__(self, kw) 
 441          self._solver = None 
 442          self.diagnostics._errorcodes = {
 
 443                0: 'Unrecognized error code returned (see stderr output)',
 
 444              -1 : 'input is not consistent',
 
 445              -2 : 'larger nmax is needed',
 
 446               2 : 'larger nmax or maxevtpts is probably needed (error raised by solout)',
 
 447              -3 : 'step size becomes too small',
 
 448              -4 : 'the matrix is repeatedly singular (interrupted)',
 
 449              -8 : 'The solution exceeded a magbound (poor choice of initial step)'} 
 450          self.diagnostics.outputStatsInfo = {
 
 451              'last_step': 'Predicted step size of the last accepted step (useful for a subsequent call to radau).',
 
 452              'num_steps': 'Number of used steps.',
 
 453              'num_accept': 'Number of accepted steps.',
 
 454              'num_reject': 'Number of rejected steps.',
 
 455              'num_fcns': 'Number of function evaluations.',
 
 456              'num_jacs': 'Number of Jacobian evaluations.',
 
 457              'num_dec': 'Number of LU-decompositions',
 
 458              'num_subs': 'Number of forward-backward substitutions',
 
 459              'errorStatus': 'Error status on completion.'
 
 460               } 
 461  
 
 462          # currently the final four of these params are for event handling
 
 463          algparams_def = {'poly_interp': False,
 
 464                          'init_step': 0,
 
 465                          'max_step': 0,
 
 466                          'rtol': [1e-9 for i in range(self.dimension)],
 
 467                          'atol': [1e-12 for i in range(self.dimension)],
 
 468                          'fac1': 1.0,
 
 469                          'fac2': 1.2,
 
 470                          'stepLB': 0.2,
 
 471                          'stepUB': 8.0,
 
 472                          'safety': 0.9,
 
 473                          'max_pts': 10000,
 
 474                          'refine': 0,
 
 475                          'maxbisect': [], # for events
 
 476                          'maxevtpts': 1000, # for events
 
 477                          'eventInt': [], # set using setEventInterval only
 
 478                          'eventDelay': [], # set using setEventDelay only
 
 479                          'eventTol': [], # set using setEventTol only
 
 480                          'use_special': 0,
 
 481                          'specialtimes': [],
 
 482                          'check_aux': 1,
 
 483                          'extraspace': 100,
 
 484                          'verbose': 0,
 
 485                          'jac_recompute': 0.001,
 
 486                          'step_strategy': 1,
 
 487                          'index1dim': -1,
 
 488                          'index2dim': 0,
 
 489                          'index3dim': 0,
 
 490                          'DAEstructureM1': 0,
 
 491                          'DAEstructureM2': 0,
 
 492                          'hessenberg': 0,
 
 493                          'newton_start': 0,
 
 494                          'newton_stop': -1,
 
 495                          'max_newton': 7,
 
 496                          'hasJac': 0,
 
 497                          'hasJacP': 0,
 
 498                          'checkBounds': self.checklevel
 
 499                          } 
 500          for k, v in algparams_def.iteritems(): 
 501              if k not in self.algparams: 
 502                  self.algparams[k] = v 
 503          # verify that no additional keys are present in algparams, after
 
 504          # defaults are added above
 
 505          if len(self.algparams) != len(algparams_def): 
 506              raise ValueError("Invalid keys present in algparams argument: " \
 
 507                       + str(remain(self.algparams.keys(),algparams_def.keys()))) 
 508          # Check for non-constant mass matrix
 
 509          if self.haveMass(): 
 510              mspec = self.funcspec.auxfns['massMatrix'] 
 511              lensig = len(mspec[1]) 
 512              body_str = mspec[0][lensig:].replace('\n','') 
 513              qbody = QuantSpec('__body__', body_str, treatMultiRefs=False,
 
 514                                ignoreSpecial=['[',']','{','}']) 
 515              self._const_massmat = intersect(['Y_','t'], qbody.usedSymbols) == [] 
 516          else: 
 517              self._const_massmat = True 
 518          # File system preparations
 
 519          thisplatform = platform.system() 
 520          if thisplatform == 'Windows': 
 521              self._dllext = ".pyd" 
 522          elif thisplatform in ['Linux', 'IRIX', 'Solaris', 'SunOS', 'MacOS', 'Darwin']: 
 523              self._dllext = '.so' 
 524          else: 
 525              print "Shared library extension not tested on this platform." 
 526              print "If this process fails please report the errors to the" 
 527              print "developers." 
 528              self._dllext = '.so' 
 529          self._compilation_tempdir = os.path.join(os.getcwd(),
 
 530                                                        "radau5_temp") 
 531          if not os.path.isdir(self._compilation_tempdir): 
 532              try: 
 533                  assert not os.path.isfile(self._compilation_tempdir), \
 
 534                       "A file already exists with the same name" 
 535                  os.mkdir(self._compilation_tempdir) 
 536              except: 
 537                  print "Could not create compilation temp directory " + \
 
 538                        self._compilation_tempdir 
 539                  raise 
 540          self._compilation_sourcedir = os.path.join(_pydstool_path,"integrator") 
 541          self._vf_file = self.name+"_vf.c" 
 542          self._vf_filename_ext = "_"+self._vf_file[:-2] 
 543          self._prepareEventSpecs() 
 544          if not (os.path.isfile(os.path.join(os.getcwd(),
 
 545                                  "radau5"+self._vf_filename_ext+".py")) and \
 
 546                  os.path.isfile(os.path.join(os.getcwd(),
 
 547                                  "_radau5"+self._vf_filename_ext+self._dllext))): 
 548              if not nobuild: 
 549                  self.makeLibSource() 
 550                  self.compileLib() 
 551              else: 
 552                  print "Build the library using the makeLib method, or in " 
 553                  print "stages using the makeLibSource and compileLib methods." 
 554          self._inputVarList = [] 
 555          self._inputTimeList = []

 556  
 
 557  
 


558 -    def forceLibRefresh(self):


559          """forceLibRefresh should be called after event contents are changed,
 
 560          or alterations are made to the right-hand side of the ODEs.
 
 561  
 
 562          Currently this function does NOT work!""" 
 563  
 
 564          # (try to) free radau module from namespace
 
 565          delfiles = True 
 566          self._solver = None 
 567          try: 
 568              del(sys.modules["_radau5"+self._vf_filename_ext]) 
 569              del(sys.modules["radau5"+self._vf_filename_ext]) 
 570  ##            del(self._integMod)
 
 571          except NameError: 
 572              # modules weren't loaded, so nothing to do
 
 573              delfiles = False 
 574          if delfiles: 
 575              gc.collect() 
 576              # still not able to delete these files!!!!! Argh!
 
 577  ##            if os.path.isfile(os.path.join(os.getcwd(),
 
 578  ##                                    "radau5"+self._vf_filename_ext+".py")):
 
 579  ##                os.remove(os.path.join(os.getcwd(),
 
 580  ##                                    "radau5"+self._vf_filename_ext+".py"))
 
 581  ##            if os.path.isfile(os.path.join(os.getcwd(),
 
 582  ##                                 "_radau5"+self._vf_filename_ext+self._dllext)):
 
 583  ##                os.remove(os.path.join(os.getcwd(),
 
 584  ##                                 "_radau5"+self._vf_filename_ext+self._dllext))
 
 585          print "Cannot rebuild library without restarting session. Sorry." 
 586          print "Try asking the Python developers to make a working module" 
 587          print "unimport function!"

 588  ##        self.makeLibSource()
 
 589  
 
 590  
 


591 -    def _prepareEventSpecs(self):


592          eventActive = [] 
 593          eventTerm = [] 
 594          eventDir = [] 
 595          eventDelay = [] 
 596          eventTol = [] 
 597          maxbisect = [] 
 598          eventInt = [] 
 599          # convert event specs (term, active, etc.) into integparam specs
 
 600          self._eventNames = self.eventstruct.sortedEventNames() 
 601          for evname in self._eventNames: 
 602              ev = self.eventstruct.events[evname] 
 603              assert isinstance(ev, LowLevelEvent), ("Radau can only "
 
 604                                                  "accept low level events") 
 605          # if event 'precise' flags set to False then set their tolerances
 
 606          # to be > max_step
 
 607          maxstep = self.algparams['max_step'] 
 608          for evname in self._eventNames: 
 609              ev = self.eventstruct.events[evname] 
 610              eventActive.append(int(ev.activeFlag)) 
 611              eventTerm.append(int(ev.termFlag)) 
 612              eventDir.append(ev.dircode) 
 613              eventInt.append(ev.eventinterval) 
 614              eventDelay.append(ev.eventdelay) 
 615              if ev.preciseFlag: 
 616                  eventTol.append(ev.eventtol) 
 617                  maxbisect.append(ev.bisectlimit) 
 618              else: 
 619                  eventTol.append(maxstep*1.5) 
 620                  maxbisect.append(1) 
 621          self.algparams['eventTol'] = eventTol 
 622          self.algparams['eventDelay'] = eventDelay 
 623          self.algparams['eventInt'] = eventInt 
 624          self.algparams['maxbisect'] = maxbisect 
 625          self.algparams['eventActive'] = eventActive 
 626          self.algparams['eventTerm'] = eventTerm 
 627          self.algparams['eventDir'] = eventDir

 628  
 
 629  
 


630 -    def makeLib(self, libsources=[], libdirs=[], include=[]):


631          """makeLib calls makeLibSource and then the compileLib method.
 
 632          To postpone compilation of the source to a DLL, call makelibsource()
 
 633          separately.""" 
 634          self.makeLibSource(include) 
 635          self.compileLib(libsources, libdirs)

 636  
 
 637  
 


638 -    def makeLibSource(self, include=[]):


639          """makeLibSource generates the C source for the vector field specification.
 
 640          It should be called only once per vector field.""" 
 641  
 
 642          # Make vector field (and event) file for compilation
 
 643          assert isinstance(include, list), "includes must be in form of a list" 
 644          # codes for library types (default is USERLIB, since compiler will look in standard library d
 
 645          STDLIB = 0 
 646          USERLIB = 1 
 647          libinclude = dict([('Python.h', STDLIB), ('math.h', STDLIB), ('stdio.h', STDLIB), ('stdlib.h', STDLIB),
 
 648                        ('string.h', STDLIB), ('vfield.h', USERLIB), ('events.h', USERLIB),
 
 649                        ('signum.h', USERLIB), ('maxmin.h', USERLIB)]) 
 650          include_str = '' 
 651          for libstr, libtype in libinclude.iteritems(): 
 652              if libtype == STDLIB: 
 653                  quoteleft = '<' 
 654                  quoteright = '>' 
 655              else: 
 656                  quoteleft = '"' 
 657                  quoteright = '"' 
 658              include_str += "#include " + quoteleft + libstr + quoteright + "\n" 
 659          if include != []: 
 660              assert isUniqueSeq(include), "list of library includes must not contain repeats" 
 661              for libstr in include: 
 662                  if libstr in libinclude: 
 663                      # don't repeat libraries
 
 664                      print "Warning: library '" + libstr + "' already appears in list"\
 
 665                            + " of imported libraries" 
 666                  else: 
 667                      include_str += "#include " + '"' + libstr + '"\n' 
 668          allfilestr = "/*  Vector field function and events for Radau integrator.\n" \
 
 669              + "  This code was automatically generated by PyDSTool, but may be modified " \
 
 670              + "by hand. */\n\n" + include_str + """
 
 671  extern double *gICs;
 
 672  extern double **gBds;
 
 673  extern double globalt0;
 
 674  
 
 675  static double pi = 3.1415926535897931;
 
 676  
 
 677  double signum(double x)
 
 678  {
 
 679    if (x<0) {
 
 680      return -1;
 
 681    }
 
 682    else if (x==0) {
 
 683      return 0;
 
 684    }
 
 685    else if (x>0) {
 
 686      return 1;
 
 687    }
 
 688    else {
 
 689      /* must be that x is Not-a-Number */
 
 690      return x;
 
 691    }
 
 692  }
 
 693  
 
 694  """ 
 695          pardefines = "" 
 696          vardefines = "" 
 697          auxvardefines = "" 
 698          inpdefines = "" 
 699          # sorted version of var, par, and input names
 
 700          vnames = self._var_ixmap 
 701          pnames = self.funcspec.pars 
 702          inames = self.funcspec.inputs 
 703          pnames.sort() 
 704          inames.sort() 
 705          for i in xrange(self.numpars): 
 706              p = pnames[i] 
 707              # add to defines
 
 708              pardefines += self.funcspec._defstr+" "+p+"\tp_["+str(i)+"]\n" 
 709          for i in xrange(self.dimension): 
 710              v = vnames[i] 
 711              # add to defines
 
 712              vardefines += self.funcspec._defstr+" "+v+"\tY_["+str(i)+"]\n" 
 713          for i, v in enumerate(self.funcspec.auxvars): 
 714              auxvardefines += self.funcspec._defstr+" "+v+"\t("+self.funcspec._auxdefs_parsed[v]+")\n" 
 715          for i in xrange(len(self.funcspec.inputs)): 
 716              inp = inames[i] 
 717              # add to defines
 
 718              inpdefines += self.funcspec._defstr+" "+inp+"\txv_["+str(i)+"]\n" 
 719          allfilestr += "\n/* Variable, aux variable, parameter, and input definitions: */ \n" \
 
 720                        + pardefines + vardefines + auxvardefines + inpdefines + "\n" 
 721          # preprocess event code
 
 722          allevs = "" 
 723          if self._eventNames == []: 
 724              numevs = 0 
 725          else: 
 726              numevs = len(self._eventNames) 
 727          for evname in self._eventNames: 
 728              ev = self.eventstruct.events[evname] 
 729              evfullfn = "" 
 730              assert isinstance(ev, LowLevelEvent), ("Radau can only "
 
 731                                                  "accept low level events") 
 732              evsig = ev._LLreturnstr + " " + ev.name + ev._LLargstr 
 733              assert ev._LLfuncstr.index(';') > 1, ("Event function code "
 
 734                      "error: Have you included a ';' character at the end of"
 
 735                                              "your 'return' statement?") 
 736              fbody = ev._LLfuncstr 
 737              # check fbody for calls to user-defined aux fns
 
 738              # and add hidden p argument
 
 739              if self.funcspec.auxfns: 
 740                  fbody_parsed = addArgToCalls(fbody,
 
 741                                          self.funcspec.auxfns.keys(),
 
 742                                          "p_, wk_, xv_") 
 743                  if 'initcond' in self.funcspec.auxfns: 
 744                      # convert 'initcond(x)' to 'initcond("x")' for
 
 745                      # compatibility with C syntax
 
 746                      fbody_parsed = wrapArgInCall(fbody_parsed,
 
 747                                          'initcond', '"') 
 748              else: 
 749                  fbody_parsed = fbody 
 750              evbody = " {\n" + fbody_parsed + "\n}\n\n" 
 751              allevs += evsig + evbody 
 752              allfilestr += evsig + ";\n" 
 753          # add signature for auxiliary functions
 
 754          if self.funcspec.auxfns: 
 755              allfilestr += "\n" 
 756              for finfo in self.funcspec.auxfns.values(): 
 757                  allfilestr += finfo[1] + ";\n" 
 758          assignEvBody = "" 
 759          for evix in range(numevs): 
 760              assignEvBody += "events[%d] = &%s;\n"%(evix,self._eventNames[evix]) 
 761          allfilestr += "\nint N_EVENTS = " + str(numevs) + ";\nvoid assignEvents(" \
 
 762                + "EvFunType *events){\n " + assignEvBody  \
 
 763                + "\n}\n\nvoid auxvars(unsigned, unsigned, double, double*, double*, " \
 
 764                + "double*, unsigned, double*, unsigned, double*);\n" \
 
 765                + """void jacobian(unsigned, unsigned, double, double*, double*, double**, unsigned, double*, unsigned, double*);
 
 766  void jacobianParam(unsigned, unsigned, double, double*, double*, double**, unsigned, double*, unsigned, double*);
 
 767  """ 
 768          if self.funcspec.auxvars == []: 
 769              allfilestr += "int N_AUXVARS = 0;\n\n\n" 
 770          else: 
 771              allfilestr += "int N_AUXVARS = " + str(len(self.funcspec.auxvars)) \
 
 772                         + ";\n\n\n" 
 773          if self.funcspec.inputs == []: 
 774              allfilestr += "int N_EXTINPUTS = 0;\n\n\n" 
 775          else: 
 776              allfilestr += "int N_EXTINPUTS = " + str(len(self.funcspec.inputs)) \
 
 777                         + ";\n\n\n" 
 778          allfilestr += self.funcspec.spec[0] + "\n\n" 
 779          if self.funcspec.auxfns: 
 780              for fname, finfo in self.funcspec.auxfns.iteritems(): 
 781                  fbody = finfo[0] 
 782                  # subs _p into auxfn-to-auxfn calls (but not to the signature)
 
 783                  fbody_parsed = addArgToCalls(fbody,
 
 784                                          self.funcspec.auxfns.keys(),
 
 785                                          "p_, wk_, xv_", notFirst=fname) 
 786                  if 'initcond' in self.funcspec.auxfns: 
 787                      # convert 'initcond(x)' to 'initcond("x")' for
 
 788                      # compatibility with C syntax, but don't affect the
 
 789                      # function signature!
 
 790                      fbody_parsed = wrapArgInCall(fbody_parsed,
 
 791                                          'initcond', '"', notFirst=True) 
 792                  allfilestr += "\n" + fbody_parsed + "\n\n" 
 793          # add auxiliary variables (shell of the function always present)
 
 794          # add event functions
 
 795          allfilestr += self.funcspec.auxspec[0] + allevs 
 796          # if jacobians or mass matrix not present, fill in dummy
 
 797          if not self.haveMass(): 
 798              allfilestr += """
 
 799  void massMatrix(unsigned n_, unsigned np_, double t, double *Y_, double *p_, double **f_, unsigned wkn_, double *wk_, unsigned xvn_, double *xv_) {
 
 800  }
 
 801  """ 
 802          if not self.haveJacobian(): 
 803              allfilestr += """
 
 804  void jacobian(unsigned n_, unsigned np_, double t, double *Y_, double *p_, double **f_, unsigned wkn_, double *wk_, unsigned xvn_, double *xv_) {
 
 805  }
 
 806  """ 
 807          if not self.haveJacobian_pars(): 
 808              allfilestr += """
 
 809  void jacobianParam(unsigned n_, unsigned np_, double t, double *Y_, double *p_, double **f_, unsigned wkn_, double *wk_, unsigned xvn_, double *xv_) {
 
 810  }
 
 811  """ #+ "\n/* Variable and parameter substitutions undefined:*/\n" + parundefines + varundefines + "\n" 
 812          # write out C file
 
 813          vffile = os.path.join(self._compilation_tempdir, self._vf_file) 
 814          try: 
 815              file = open(vffile, 'w') 
 816              file.write(allfilestr) 
 817              file.close() 
 818          except IOError, e: 
 819              print "Error opening file "+self._vf_file+" for writing" 
 820              raise IOError, e

 821  
 
 822  
 


823 -    def compileLib(self, libsources=[], libdirs=[]):


824          """compileLib generates a python extension DLL with integrator and vector
 
 825          field compiled and linked.
 
 826  
 
 827          libsources list allows additional library sources to be linked.
 
 828          libdirs list allows additional directories to be searched for
 
 829            precompiled libraries.""" 
 830  
 
 831          if os.path.isfile(os.path.join(os.getcwd(),
 
 832                                  "_radau5"+self._vf_filename_ext+self._dllext)): 
 833              # then DLL file already exists and we can't overwrite it at this
 
 834              # time
 
 835              proceed = False 
 836              print "\n" 
 837              print "-----------------------------------------------------------" 
 838              print "Present limitation of Python: Cannot rebuild library" 
 839              print "without exiting Python and deleting the shared library" 
 840              print "   " + str(os.path.join(os.getcwd(),
 
 841                                  "_radau5"+self._vf_filename_ext+self._dllext)) 
 842              print "by hand! If you made any changes to the system you should" 
 843              print "not proceed with running the integrator until you quit" 
 844              print "and rebuild." 
 845              print "-----------------------------------------------------------" 
 846              print "\n" 
 847          else: 
 848              proceed = True 
 849          if not proceed: 
 850              print "Did not compile shared library." 
 851              return 
 852          if self._solver is not None: 
 853              self.forceLibRefresh() 
 854          vffile = os.path.join(self._compilation_tempdir, self._vf_file) 
 855          try: 
 856              ifacefile_orig = open(os.path.join(self._compilation_sourcedir,
 
 857                                                 "radau5.i"), 'r') 
 858              ifacefile_copy = open(os.path.join(self._compilation_tempdir,
 
 859                                         "radau5_"+self._vf_file[:-2]+".i"), 'w') 
 860              firstline = ifacefile_orig.readline() 
 861              ifacefile_copy.write('%module radau5_'+self._vf_file[:-2]+'\n') 
 862              iffilestr = ifacefile_orig.read() 
 863              ifacefile_copy.write(iffilestr) 
 864              ifacefile_orig.close() 
 865              ifacefile_copy.close() 
 866          except IOError: 
 867              print "radau5.i copying error in radau compilation directory" 
 868              raise 
 869          swigfile = os.path.join(self._compilation_tempdir,
 
 870                                  "radau5"+self._vf_filename_ext+".i") 
 871          radwrapfile = os.path.join(self._compilation_sourcedir, "radau5mod.c") 
 872          if self._const_massmat: 
 873              radfile = os.path.join(self._compilation_sourcedir, "radau5.f") 
 874          else: 
 875              # version for non-constant mass matrices
 
 876              radfile = os.path.join(self._compilation_sourcedir, "radau5v.f") 
 877          dc_lapackfile = os.path.join(self._compilation_sourcedir, "dc_lapack.f") 
 878          lapackfile = os.path.join(self._compilation_sourcedir, "lapack.f") 
 879          lapackcfile = os.path.join(self._compilation_sourcedir, "lapackc.f") 
 880          integfile = os.path.join(self._compilation_sourcedir, "integration.c") 
 881          interfacefile = os.path.join(self._compilation_sourcedir, "interface.c") 
 882          eventfile = os.path.join(self._compilation_sourcedir, "eventFinding.c") 
 883          memfile = os.path.join(self._compilation_sourcedir, "memory.c") 
 884          # The following if statement attempts to avoid recompiling the SWIG wrapper
 
 885          # if the files mentioned already exist, because in principle the SWIG interface
 
 886          # only needs compiling once. But this step doesn't seem to work yet.
 
 887          # Instead, it seems that SWIG always gets recompiled with everything else
 
 888          # (at least on Win32). Maybe the list of files is incorrect...
 
 889          if not (all([os.path.isfile(os.path.join(self._compilation_tempdir,
 
 890                     sf)) for sf in ['radau5'+self._vf_filename_ext+'_wrap.o',
 
 891                                     'lib_radau5'+self._vf_filename_ext+'.a',
 
 892                                     'radau5'+self._vf_filename_ext+'.py',
 
 893                                     '_radau5'+self._vf_filename_ext+'.def']])): 
 894              modfilelist = [swigfile] 
 895          else: 
 896              modfilelist = [] 
 897  ##        modfilelist = [swigfile]
 
 898          modfilelist.extend([radwrapfile, vffile, integfile, eventfile,
 
 899                             interfacefile, memfile]) 
 900          modfilelist.extend(libsources) 
 901          fortfilelist=[radfile, lapackcfile, dc_lapackfile, lapackfile] 
 902          # would like temp dir to be '/' but numpy_distutils cannot build the
 
 903          # radau library in that configuration
 
 904          script_args = ['-q', 'build', '--build-lib=.',#+os.getcwd(),
 
 905                   '-tradau5_temp',#+self._compilation_tempdir,
 
 906                   '--build-base=radau5_temp',#+self._compilation_tempdir] #,
 
 907                   '--build-platlib=radau5_temp', '--build-purelib=radau5_temp'] 
 908          if self._compiler != '': 
 909              script_args.append('-c'+str(self._compiler)) 
 910  
 
 911          # include directories for libraries
 
 912          narraydir = npy.get_numarray_include() 
 913          npydir = npy.get_include() 
 914  
 
 915          incdirs = [npydir, narraydir, os.getcwd(), self._compilation_tempdir,
 
 916                     self._compilation_sourcedir] 
 917          incdirs.extend(libdirs) 
 918          radlibdirs = [self._compilation_sourcedir, self._compilation_tempdir,
 
 919                         os.getcwd()] 
 920          # Use distutils to perform the compilation of the selected files
 
 921          rout.start()    # redirect stdout 
 922          try: 
 923              distobject = setup(name = "Radau5 integrator",
 
 924                    author = "PyDSTool (automatically generated)",
 
 925                    script_args = script_args,
 
 926                    ext_modules = [Extension("_radau5"+self._vf_filename_ext,
 
 927                                   sources=modfilelist,
 
 928                                   include_dirs=incdirs,
 
 929                                   library_dirs=radlibdirs,
 
 930                                   libraries=['radau5'],
 
 931                                   extra_compile_args=['-w', '-D__RADAU__', '-m32'],
 
 932                                   extra_link_args=['-w', '-m32'])],
 
 933  #                  library_dirs=radlibdirs,
 
 934                    libraries=[('radau5',{'sources': fortfilelist,
 
 935                                'library_dirs': radlibdirs+['./']})]) 
 936          except: 
 937              rout.stop() 
 938              print "\nError occurred in generating Radau system..." 
 939              print sys.exc_info()[0], sys.exc_info()[1] 
 940              raise RuntimeError 
 941          rout.stop()    # restore stdout 
 942          # Attempt to unload module through a shutdown() function being
 
 943          # added to the SWIG module file. But it didn't work!
 
 944  ##        try:
 
 945  ##            modfilepy = open(os.path.join(self._compilation_tempdir,
 
 946  ##                                    "radau5"+self._vf_filename_ext+".py"), 'a')
 
 947  ##            extfilename = "_radau5"+self._vf_filename_ext
 
 948  ##            modfilepy.write("""# The following addition made by PyDSTool:
 
 949  ##def shutdown():
 
 950  ##    import sys
 
 951  ##    del sys.modules['""" + extfilename + """']
 
 952  ##            """)
 
 953  ####    del """ + extfilename + """
 
 954  ####    del new_doubleArray
 
 955  ####    del delete_doubleArray
 
 956  ####    del doubleArray_getitem
 
 957  ####    del doubleArray_setitem
 
 958  ####    del new_intArray
 
 959  ####    del delete_intArray
 
 960  ####    del intArray_getitem
 
 961  ####    del intArray_setitem
 
 962  ####    del Integrate
 
 963  ##            modfilepy.close()
 
 964  ##        except IOError:
 
 965  ##            print "radau5.py modifying error in radau temp compilation " \
 
 966  ##                  + "directory"
 
 967  ##            raise
 
 968          try: 
 969              # move library files into the user's CWD
 
 970              distdestdir = distutil_destination() 
 971              if swigfile in modfilelist or not \
 
 972                     os.path.isfile(os.path.join(self._compilation_tempdir,
 
 973                                  "radau5"+self._vf_filename_ext+".py")): 
 974                  # temporary hack to fix numpy_distutils bug
 
 975                  shutil.move(os.path.join(os.getcwd(),
 
 976                                           self._compilation_tempdir, distdestdir,
 
 977                                           "radau5_temp",
 
 978                                           "radau5"+self._vf_filename_ext+".py"),
 
 979                              os.path.join(os.getcwd(),
 
 980                                           "radau5"+self._vf_filename_ext+".py")) 
 981          except: 
 982              print "\nError occurred in generating Radau system" 
 983              print "(while moving library extension modules to CWD)" 
 984              #print sys.exc_info()[0], sys.exc_info()[1]
 
 985              raise #RuntimeError

 986  
 
 987      # doesn't work!
 
 988  #    def _ensureLoaded(self, modname):
 
 989  ##        if modname in sys.modules:
 
 990  ##            _integMod = reload(sys.modules[modname])
 
 991  ##        else:
 
 992  #        try:
 
 993  #            _integMod = __import__(modname, globals())
 
 994  #        except:
 
 995  #            print "Error in importing compiled vector field and integrator."
 
 996  #            print "Did you compile the RHS C code?"
 
 997  #            raise
 
 998  #        # Initialize integrator
 
 999  #        assert 'Integrate' in dir(_integMod), \
 
1000  #               "radau library does not contain Integrate()"
 
1001  #        return _integMod
 
1002  
 
1003  
 


1004 -    def compute(self, trajname, dirn='f', ics=None):


1005          continue_integ = ODEsystem.prepDirection(self, dirn) 
1006  #        _integMod = __import__("radau5"+self._vf_filename_ext, globals())
 
1007          if ics is not None: 
1008              self.set(ics=ics) 
1009          self.validateICs() 
1010          self.diagnostics.clearWarnings() 
1011          self.diagnostics.clearErrors() 
1012          if isinstance(self.algparams['rtol'], list): 
1013              if len(self.algparams['rtol']) != self.dimension: 
1014                  raise ValueError('rtol list must have same length as phase dimension') 
1015          else: 
1016              rtol = self.algparams['rtol'] 
1017              self.algparams['rtol'] = [rtol for i in range(self.dimension)] 
1018          if isinstance(self.algparams['atol'], list): 
1019              if len(self.algparams['atol']) != self.dimension: 
1020                  raise ValueError('atol list must have same length as phase dimension') 
1021          else: 
1022              atol = self.algparams['atol'] 
1023              self.algparams['atol'] = [atol for i in range(self.dimension)] 
1024          anames = self.funcspec.auxvars 
1025          # Check i.c.'s are well defined (finite)
 
1026          self.checkInitialConditions() 
1027          self.setEventICs(self.initialconditions, self.globalt0) 
1028          # update event params in case changed since last run
 
1029          self._prepareEventSpecs() 
1030          # Main integration
 
1031          t0 = self.indepvariable.depdomain[0] 
1032          t1 = self.indepvariable.depdomain[1] 
1033          plist = sortedDictValues(self.pars) 
1034          self.algparams['hasJac'] = self.haveJacobian() 
1035          self.algparams['hasJacP'] = self.haveJacobian_pars() 
1036          self._ensure_solver() 
1037          if self._dircode == 1: 
1038              tbegin = t0 
1039              tend = t1 
1040          elif self._dircode == -1: 
1041              # radau does reverse time integration simply by switching t0 and t1
 
1042              tbegin = t1 
1043              tend = t0 
1044          if len(self.algparams['specialtimes'])>0: 
1045              use_special = self.algparams['use_special'] 
1046          else: 
1047              use_special = 0 
1048          bounds = [[],[]]  # lower, then upper 
1049          for v in self.funcspec.vars: 
1050              bds = self.xdomain[v] 
1051              try: 
1052                  bounds[0].append(bds[0]) 
1053                  bounds[1].append(bds[1]) 
1054              except TypeError: 
1055                  print v, type(bds), bds 
1056                  print self.xdomain 
1057                  raise 
1058          for p in self.funcspec.pars: 
1059              bds = self.pdomain[p] 
1060              try: 
1061                  bounds[0].append(bds[0]) 
1062                  bounds[1].append(bds[1]) 
1063              except TypeError: 
1064                  print type(bds), bds 
1065                  raise 
1066          if continue_integ: 
1067              x0 = self._solver.lastPoint 
1068              # overwrite t0 from self.indepvariable.domain, but use its t1
 
1069              tbegin = self._solver.lastTime 
1070              if abs(self._solver.lastStep) < abs(self.algparams['init_step']): 
1071                  self.algparams['init_step'] = self._solver.lastStep 
1072              if abs(t1-tbegin) < abs(self.algparams['init_step']): 
1073                  raise ValueError("Integration end point too close to initial "
 
1074                                   "point") 
1075  #            if self.inputs and self._extInputsChanged:
 
1076  #                self._extInputsChanged = False
 
1077  #                self._solver.setContParams(tend, plist,
 
1078  #                                           use_special,
 
1079  #                                           self.algparams['verbose'],
 
1080  #                                           True, deepcopy(self._inputVarList),
 
1081  #                                           deeppcopy(self._inputTimeList))
 
1082          else: 
1083              if self._solver.numRuns > 0: 
1084                  self._solver.clearAll() 
1085              x0 = sortedDictValues(self.initialconditions, self.funcspec.vars) 
1086              self._solver.setInteg(maxpts=self.algparams['max_pts'],
 
1087                  rtol=self.algparams['rtol'], atol=self.algparams['atol']) 
1088              self._solver.setRunParams(ic=x0, params=plist,
 
1089                                    t0=tbegin, tend=tend, gt0=self.globalt0,
 
1090                                    refine=self.algparams['refine'],
 
1091                                    specTimes=self.algparams['specialtimes'],
 
1092                                    bounds=bounds) 
1093          if self.inputs: 
1094              # self._extInputsChanged if global t0 changed so that can
 
1095              # adjust times given to the integrator (it is blind to global t0
 
1096              # when accesses input variable times)
 
1097              self._ensure_inputs(self._extInputsChanged) 
1098          # hinit only set if not continue_integ
 
1099          if len(anames)>0: 
1100              check_aux = self.algparams['check_aux'] 
1101          else: 
1102              check_aux = 0 
1103          if self.algparams['max_step'] == 0: 
1104              max_step = abs(tend-tbegin) 
1105          else: 
1106              max_step = self.algparams['max_step'] 
1107          init_step = self.algparams['init_step'] 
1108          if self._dircode == 1: 
1109              if init_step < 0: 
1110                  init_step = -init_step 
1111              if max_step < 0: 
1112                  max_step = -max_step 
1113          else: 
1114              if init_step > 0: 
1115                  init_step = -init_step 
1116              if max_step > 0: 
1117                  max_step = -max_step 
1118          if continue_integ: 
1119              # record needed for bounds checking and truncation
 
1120              old_highest_ix = self._solver.points.shape[1] 
1121              alltData, X, A, Stats, H, Err, Evtimes, \
 
1122                  Evpoints = self._solver.Continue(tend, plist,
 
1123                                    use_special, self.algparams['verbose'],
 
1124                                    self._extInputsChanged,
 
1125                                    deepcopy(self._inputVarList),
 
1126                                    deepcopy(self._inputTimeList), bounds) 
1127          else: 
1128              old_highest_ix = 0 
1129              self._solver.setEvents(eventActive=self.algparams['eventActive'],
 
1130                              eventTerm=self.algparams['eventTerm'],
 
1131                              eventDir=self.algparams['eventDir'],
 
1132                              eventDelay=self.algparams['eventDelay'],
 
1133                              eventInt=self.algparams['eventInt'],
 
1134                              eventTol=self.algparams['eventTol'],
 
1135                              maxevtpts=self.algparams['maxevtpts'],
 
1136                              maxbisect=self.algparams['maxbisect']) 
1137              alltData, X, A, Stats, H, Err, Evtimes, \
 
1138                  Evpoints = self._solver.Run(init_step,
 
1139                                      max_step,
 
1140                                      check_aux,
 
1141                                      use_special,
 
1142                                      self.algparams['verbose'],
 
1143                                      self.algparams['safety'],
 
1144                                      self.algparams['jac_recompute'],
 
1145                                      self.algparams['newton_stop'],
 
1146                                      self.algparams['fac1'],
 
1147                                      self.algparams['fac2'],
 
1148                                      self.algparams['stepLB'],
 
1149                                      self.algparams['stepUB'],
 
1150                                      self.algparams['hessenberg'],
 
1151                                      self.algparams['max_newton'],
 
1152                                      self.algparams['newton_start'],
 
1153                                      self.algparams['index1dim'],
 
1154                                      self.algparams['index2dim'],
 
1155                                      self.algparams['index3dim'],
 
1156                                      self.algparams['step_strategy'],
 
1157                                      self.algparams['DAEstructureM1'],
 
1158                                      self.algparams['DAEstructureM2'],
 
1159                                      self.haveJacobian(),
 
1160                                      self.haveMass()) 
1161          self._extInputsChanged = False    # reset this now 
1162          self.diagnostics.outputStats = {'last_step': H,
 
1163                              'last_time': self._solver.lastTime,
 
1164                              'last_point': self._solver.lastPoint,
 
1165                              'num_fcns': Stats[0],
 
1166                              'num_jacs': Stats[1],
 
1167                              'num_steps': Stats[2],
 
1168                              'num_accept': Stats[3],
 
1169                              'num_reject': Stats[4],
 
1170                              'num_dec': Stats[5],
 
1171                              'num_subs': Stats[6],
 
1172                              'errorStatus': Err
 
1173                              } 
1174          if self._dircode == -1: 
1175              # reverse the array object (no reverse method!)
 
1176              alltData = alltData[::-1] 
1177              X = X[:,::-1] 
1178              if anames != []: 
1179                  A = A[:,::-1] 
1180          xnames = self._var_ixmap 
1181          # Package up computed trajectory in Variable variables
 
1182          # Add external inputs warnings to self.diagnostics.warnings, if any
 
1183  ##        for f in inputVarList:
 
1184  ##            for winfo in f.diagnostics.warnings:
 
1185  ##                self.diagnostics.warnings.append((W_NONTERMSTATEBD,
 
1186  ##                                     (winfo[0], f.name, winfo[1],
 
1187  ##                                      f.depdomain)))
 
1188          eventslist = self.eventstruct.query(['lowlevel', 'active']) 
1189          termevents = self.eventstruct.query(['term'], eventslist) 
1190          if self._eventNames != []: 
1191              # build self.diagnostics.warnings because events happened --
 
1192              # and keep a record of which times terminal events happened because
 
1193              # Model.py's event handling procedure assumes multiple events
 
1194              # happening at one time are listed in one warning
 
1195              termevtimes = {} 
1196              nontermevtimes = {} 
1197              try: 
1198                  for evix in range(len(self._eventNames)): 
1199                      if Evpoints[evix] is None: 
1200                          continue 
1201                      evname = self._eventNames[evix] 
1202                      numevs = len(Evtimes[evix]) 
1203                      if self.algparams['eventTerm'][evix]: 
1204                          if numevs > 1: 
1205                              print "Event info:", Evpoints, Evtimes 
1206                          assert numevs <= 1, ("Internal error: more than one "
 
1207                                           "terminal event of same type found") 
1208                          # For safety, we should assert that this event
 
1209                          # also appears in termevents, but we don't
 
1210                          if Evtimes[evix][0] in termevtimes.keys(): 
1211                              # append event name to this warning
 
1212                              warning_ix = termevtimes[Evtimes[evix][0]] 
1213                              self.diagnostics.warnings[warning_ix][1][1].append(evname) 
1214                          else: 
1215                              # make new termevtime entry for the new warning
 
1216                              termevtimes[Evtimes[evix][0]] = \
 
1217                                         len(self.diagnostics.warnings) 
1218                              self.diagnostics.warnings.append((W_TERMEVENT,
 
1219                                               (Evtimes[evix][0],
 
1220                                               [evname]))) 
1221                      else: 
1222                          for ev in range(numevs): 
1223                              if Evtimes[evix][ev] in nontermevtimes.keys(): 
1224                                  # append event name to this warning
 
1225                                  warning_ix = nontermevtimes[Evtimes[evix][ev]] 
1226                                  self.diagnostics.warnings[warning_ix][1][1].append(evname) 
1227                              else: 
1228                                  # make new nontermevtime entry for the new warning
 
1229                                  nontermevtimes[Evtimes[evix][ev]] = \
 
1230                                                      len(self.diagnostics.warnings) 
1231                                  self.diagnostics.warnings.append((W_NONTERMEVENT,
 
1232                                                   (Evtimes[evix][ev],
 
1233                                                    [evname]))) 
1234              except IndexError: 
1235                  print "Events returned from integrator are the wrong size." 
1236                  print "  Did you change the system and not refresh the C " \
 
1237                        + "library using the forcelibrefresh() method?" 
1238                  raise 
1239          termcount = 0 
1240          for (w,i) in self.diagnostics.warnings: 
1241              if w == W_TERMEVENT or w == W_TERMSTATEBD: 
1242                  if termcount > 0: 
1243                      raise ValueError("Internal error: more than one terminal "
 
1244                                       "event found") 
1245                  termcount += 1 
1246          # post-process check of variable bounds (if defined and algparams['checkBounds'] True)
 
1247          if self._dircode > 0: 
1248              compare = operator.lt 
1249              last_ix = Inf 
1250          else: 
1251              compare = operator.gt 
1252              last_ix = -Inf 
1253          highest_ix = X.shape[1]-1 
1254          last_t = Inf 
1255          if self.algparams['checkBounds'] > 0: 
1256              # temp storage for repeatedly used object attributes (for lookup efficiency)
 
1257              depdomains = dict(zip(range(self.dimension),
 
1258                              [self.variables[xn].depdomain for xn in xnames])) 
1259              offender_ix = None 
1260              for xi in xrange(self.dimension): 
1261                  if not any(depdomains[xi].isfinite()): 
1262                      # no point in checking when the bounds are +/- infinity
 
1263                      continue 
1264                  next_last_ix = array_bounds_check(X[xi][old_highest_ix:],
 
1265                                      depdomains[xi], self._dircode) + old_highest_ix 
1266                  if compare(next_last_ix, last_ix): 
1267                      # won't count as truncating unless the following checks
 
1268                      # hold
 
1269                      last_ix = next_last_ix 
1270                      offender_ix = xi 
1271              if not isfinite(last_ix) and last_ix < 0: 
1272                  # only use +Inf hereon to flag no truncation needed
 
1273                  last_ix = Inf 
1274              elif last_ix >= 0 and last_ix < highest_ix: 
1275                  # truncate data
 
1276                  last_t = alltData[last_ix] 
1277                  print "Warning; domain bound reached (because algparams['checkBounds'] > 0)" 
1278                  self.diagnostics.warnings.append((W_TERMSTATEBD,
 
1279                                      (last_t, xnames[offender_ix],
 
1280                                       X[offender_ix, last_ix],
 
1281                                       depdomains[offender_ix].get()))) 
1282          # Create variables (self.variables contains no actual data)
 
1283          variables = copyVarDict(self.variables) 
1284          # build event pointset information (reset previous trajectory's)
 
1285          # don't include events after any truncation due to state bound violation
 
1286          self.trajevents = {} 
1287          for evix in range(len(self._eventNames)): 
1288              evname = self._eventNames[evix] 
1289              if Evpoints[evix] is None: 
1290                  self.trajevents[evname] = None 
1291              else: 
1292                  try: 
1293                      ev_a_list = [] 
1294                      for t in Evtimes[evix]: 
1295                          tix = find(alltData, t) 
1296                          ev_a_list.append(A[:,tix]) 
1297                      ev_array = concatenate((Evpoints[evix],
 
1298                                           transpose(array(ev_a_list, 'd')))) 
1299                      del ev_a_list, tix 
1300                  except TypeError: 
1301                      # A is empty
 
1302                      ev_array = Evpoints[evix] 
1303                  if last_ix >= 0 and last_ix < highest_ix: 
1304                      # don't count last_ix = -1 which is the same as highest_ix
 
1305                      last_ev_tix = npy.argmax(Evtimes[evix] >= alltData[last_ix]) 
1306                      if last_ev_tix == 0 and Evtimes[evix][0] >= last_t: 
1307                          # checks that there was actually a violation
 
1308                          # - so no events to record
 
1309                          self.trajevents[evname] = None 
1310                      else: 
1311                          # truncation needed
 
1312                          ev_array = ev_array[:, :last_ev_tix+1] 
1313                          ev_times = Evtimes[evix][:last_ev_tix+1] 
1314                          self.trajevents[evname] = Pointset({'coordnames': xnames+anames,
 
1315                                                 'indepvarname': 't',
 
1316                                                 'coordarray': ev_array,
 
1317                                                 'indepvararray': ev_times}) 
1318                  else: 
1319                      # no truncation needed
 
1320                      self.trajevents[evname] = Pointset({'coordnames': xnames+anames,
 
1321                                                 'indepvarname': 't',
 
1322                                                 'coordarray': ev_array,
 
1323                                                 'indepvararray': Evtimes[evix]}) 
1324          if last_ix >= 0 and last_ix < highest_ix: 
1325              # truncate
 
1326              X = X[:, :last_ix] 
1327              alltData = alltData[:last_ix] 
1328          try: 
1329              allxDataDict = dict(zip(xnames,X)) 
1330          except IndexError: 
1331              print "Integration returned variable values of unexpected dimensions." 
1332              print "  Did you change the system and not refresh the C library" \
 
1333                    + " using the forcelibrefresh() method?" 
1334              raise 
1335          # storage of all auxiliary variable data
 
1336          anames = self.funcspec.auxvars 
1337          try: 
1338              if anames != []: 
1339                  if last_ix < highest_ix: 
1340                      A = A[:, :last_ix] 
1341                  try: 
1342                      allaDataDict = dict(zip(anames,A)) 
1343                  except TypeError: 
1344                      print "Internal error!  Type of A: ", type(A) 
1345                      raise 
1346          except IndexError: 
1347              print "Integration returned auxiliary values of unexpected dimensions." 
1348              print "  Did you change the system and not refresh the C library" \
 
1349                    + " using the forcelibrefresh() method?" 
1350              raise 
1351          if int(Err) == 1 or (int(Err) == 2 and termcount == 1): 
1352              # output OK
 
1353              if self.algparams['poly_interp']: 
1354                  rhsfn = self._solver.Rhs 
1355                  # when Dopri can output the Rhs values alongside variable
 
1356                  # values then this won't be necessary
 
1357                  dxvals = zeros((len(alltData),self.dimension),float) 
1358                  for tix, tval in enumerate(alltData): 
1359                      # solver's Rhs function already contains the inputs so no
 
1360                      # need to recompute and provide here.
 
1361                      #i = _pollInputs(sortedDictValues(self.inputs), tval,
 
1362                      #                        self.checklevel)
 
1363                      # X is the output variable array, but rhsfn demands a list
 
1364                      dxvals[tix] = rhsfn(tval, list(X[:,tix]), plist)[0] 
1365              for xi, x in enumerate(xnames): 
1366                  if len(alltData) > 1: 
1367                      if self.algparams['poly_interp']: 
1368                          interp = PiecewisePolynomial(alltData,
 
1369                                      array([allxDataDict[x], dxvals[:,xi]]).T, 2) 
1370                      else: 
1371                          interp = interp1d(alltData, allxDataDict[x]) 
1372                      variables[x] = Variable(interp, 't', x, x) 
1373                  else: 
1374                      raise PyDSTool_ValueError("Fewer than 2 data points computed") 
1375              for a in anames: 
1376                  if len(alltData) > 1: 
1377                      variables[a] = Variable(interp1d(alltData,allaDataDict[a]),
 
1378                                               't', a, a) 
1379                  else: 
1380                      raise PyDSTool_ValueError("Fewer than 2 data points computed") 
1381              # final checks
 
1382              #self.validateSpec()
 
1383              self.defined = True 
1384              return Trajectory(trajname, variables.values(),
 
1385                                abseps=self._abseps, globalt0=self.globalt0,
 
1386                                checklevel=self.checklevel,
 
1387                                FScompatibleNames=self._FScompatibleNames,
 
1388                                FScompatibleNamesInv=self._FScompatibleNamesInv,
 
1389                                events=self.trajevents,
 
1390                                modelNames=self.name,
 
1391                                modelEventStructs=self.eventstruct) 
1392          else: 
1393              try: 
1394                  diagnost_info = self.diagnostics._errorcodes[int(Err)] 
1395              except TypeError: 
1396                  # errcode messed up from Radau
 
1397                  print "Error code: ", Err 
1398                  diagnost_info = self.diagnostics._errorcodes[0] 
1399              if self._solver.verbose: 
1400                  info(self.diagnostics.outputStats, "Output statistics") 
1401              self.defined = False 
1402              # Did the solver run out of memory?
 
1403              if (len(alltData) == self.algparams['max_pts'] or \
 
1404                  self.diagnostics.outputStats['num_steps'] >= self.algparams['max_pts']) \
 
1405                     and alltData[-1] < tend: 
1406                  print "max_pts algorithmic parameter too small: current " + \
 
1407                        "value is %i"%self.algparams['max_pts'] 
1408  #                avstep = (self.algparams['init_step']+self.diagnostics.outputStats['last_step'])/2.
 
1409                  if self.diagnostics.outputStats['last_time']-tbegin > 0: 
1410                      ms = int(round(self.algparams['max_pts'] / \
 
1411                                (self.diagnostics.outputStats['last_time'] - \
 
1412                                 tbegin)*(tend-tbegin))) 
1413                  else: 
1414                      ms = Inf 
1415                  print "(recommended value for this trajectory segment is " + \
 
1416                        "estimated to be %s (saved in diagnostics.errors attribute))"%str(ms) 
1417                  diagnost_info += " -- recommended value is %i" % ms 
1418              self.diagnostics.errors.append((E_COMPUTFAIL,
 
1419                                      (self._solver.lastTime, diagnost_info))) 
1420              raise PyDSTool_ExistError("No trajectory created")

1421  
 
1422  
 


1423 -    def Rhs(self, t, xdict, pdict=None, asarray=True):


1424          """asarray is an unused, dummy argument for compatibility with Model.Rhs""" 
1425          # don't need to convert names to FS-compatible as they sort
 
1426          # the same
 
1427          x = sortedDictValues(filteredDict(xdict, self.funcspec.vars)) 
1428          if pdict is None: 
1429              pdict = self.pars 
1430          p = sortedDictValues(pdict) 
1431          i = _pollInputs(sortedDictValues(self.inputs), t, self.checklevel) 
1432          self._ensure_solver({'params': p, 't0': 0, 'tend': 1}) 
1433          self._ensure_inputs() 
1434          return self._solver.Rhs(t, x, p+i)[0]

1435  
 
1436  
 


1437 -    def Jacobian(self, t, xdict, pdict=None, asarray=True):


1438          """asarray is an unused, dummy argument for compatibility with
 
1439          Model.Jacobian""" 
1440          if self.haveJacobian(): 
1441              x = sortedDictValues(filteredDict(xdict, self.funcspec.vars)) 
1442              if pdict is None: 
1443                  pdict = self.pars 
1444              p = sortedDictValues(pdict) 
1445              i = _pollInputs(sortedDictValues(self.inputs), t, self.checklevel) 
1446              self._ensure_solver({'params': p, 't0': 0, 'tend': 1}) 
1447              self._ensure_inputs() 
1448              return self._solver.Jacobian(t, x, p+i)[0] 
1449          else: 
1450              raise PyDSTool_ExistError("Jacobian not defined")

1451  
 
1452  
 


1453 -    def JacobianP(self, t, xdict, pdict=None, asarray=True):


1454          """asarray is an unused, dummy argument for compatibility with
 
1455          Model.JacobianP""" 
1456          if self.haveJacobian_pars(): 
1457              x = sortedDictValues(filteredDict(xdict, self.funcspec.vars)) 
1458              if pdict is None: 
1459                  pdict = self.pars 
1460              p = sortedDictValues(pdict) 
1461              i = _pollInputs(sortedDictValues(self.inputs), t, self.checklevel) 
1462              self._ensure_solver({'params': p, 't0': 0, 'tend': 1}) 
1463              self._ensure_inputs() 
1464              return self._solver.JacobianP(t, x, p+i)[0] 
1465          else: 
1466              raise PyDSTool_ExistError("Jacobian w.r.t. parameters not defined")

1467  
 
1468  
 


1469 -    def AuxVars(self, t, xdict, pdict=None, asarray=True):


1470          """asarray is an unused, dummy argument for compatibility with
 
1471          Model.AuxVars""" 
1472          x = sortedDictValues(filteredDict(xdict, self.funcspec.vars)) 
1473          if pdict is None: 
1474              pdict = self.pars 
1475          p = sortedDictValues(pdict) 
1476          i = _pollInputs(sortedDictValues(self.inputs), t, self.checklevel) 
1477          self._ensure_solver({'params': p, 't0': 0, 'tend': 1}) 
1478          self._ensure_inputs() 
1479          return self._solver.AuxFunc(t, x, p+i)[0]

1480  
 
1481  
 


1482 -    def MassMatrix(self, t, xdict, pdict=None, asarray=True):


1483          """asarray is an unused, dummy argument for compatibility with
 
1484          Model.MassMatrix""" 
1485          if self.haveMass(): 
1486              x = sortedDictValues(filteredDict(xdict, self.funcspec.vars)) 
1487              if pdict is None: 
1488                  pdict = self.pars 
1489              p = sortedDictValues(pdict) 
1490              i = _pollInputs(sortedDictValues(self.inputs), t, self.checklevel) 
1491              self._ensure_solver({'params': p, 't0': 0, 'tend': 1}) 
1492              self._ensure_inputs() 
1493              return self._solver.MassMatrix(t, x, p+i)[0] 
1494          else: 
1495              raise PyDSTool_ExistError("Mass matrix not defined")

1496  
 
1497  
 


1498 -    def _ensure_solver(self, pars=None):


1499          if self._solver is None: 
1500              x0 = sortedDictValues(filteredDict(self.initialconditions, self.funcspec.vars)) 
1501  #            _integMod = self._ensureLoaded("radau5"+self._vf_filename_ext)
 
1502              self._solver = radau("radau5"+self._vf_filename_ext,
 
1503                                   rhs=self.name, phaseDim=self.dimension,
 
1504                                   paramDim=self.numpars,
 
1505                                   nAux=len(self.funcspec.auxvars),
 
1506                                   nEvents=len(self._eventNames),
 
1507                                   nExtInputs=len(self.inputs),
 
1508                                   hasJac=self.haveJacobian(),
 
1509                                   hasJacP=self.haveJacobian_pars(),
 
1510                                   hasMass=self.haveMass(),
 
1511                                   extraSpace=self.algparams['extraspace']) 
1512              try: 
1513                  genDB.register(self) 
1514              except PyDSTool_KeyError: 
1515                  errstr = "Generator " + self.name + ": this vector field's " +\
 
1516                           "DLL is already in use" 
1517                  raise RuntimeError(errstr) 
1518              if pars is not None: 
1519                  # tend value doesn't matter
 
1520                  self._solver.setRunParams(
 
1521                                ic=sortedDictValues(filteredDict(self.initialconditions,
 
1522                                                                 self.funcspec.vars)),
 
1523                                params=pars['params'],
 
1524                                t0=pars['t0'], tend=pars['tend'],
 
1525                                gt0=self.globalt0,
 
1526                                refine=0, specTimes=[])

1527  
 


1528 -    def _ensure_inputs(self, force=False):


1529          if not self.inputs: 
1530              return 
1531          if force: 
1532              listOK = False 
1533          else: 
1534              try: 
1535                  listOK = self._inputTimest0 == self.globalt0 
1536              except AttributeError: 
1537                  # not yet defined, so proceed
 
1538                  listOK = False 
1539          if not listOK: 
1540              self._inputVarList = [] 
1541              self._inputTimeList = [] 
1542              self._inputTimest0 = self.globalt0 
1543              # inputVarList is a list of Variables or Pointsets
 
1544              for inp in sortedDictValues(self.inputs): 
1545                  if isinstance(inp, Variable): 
1546                      pts = inp.getDataPoints() 
1547                      if pts is None: 
1548                          raise TypeError("Can only pass external input Variable objects if based on"
 
1549                                          " an underlying mesh") 
1550                      else: 
1551                          tvals = copy(pts[inp.indepvarname]) 
1552                          tvals -= self.globalt0 
1553                      self._inputVarList.append(pts[inp.coordname].tolist()) 
1554                      self._inputTimeList.append(tvals.tolist()) 
1555                  elif isinstance(inp, Pointset): 
1556                      tvals = copy(inp.indepvararray) 
1557                      tvals -= self.globalt0 
1558                      self._inputVarList.append(inp[inp.coordname].tolist()) 
1559                      self._inputTimeList.append(tvals.tolist()) 
1560                  else: 
1561                      raise TypeError("Invalid type of input") 
1562          if not self._solver.initExtInputs: 
1563              self._solver.setExtInputs(True, deepcopy(self._inputVarList),
 
1564                                          deepcopy(self._inputTimeList)) 
1565          elif not listOK: 
1566              self._solver.clearExtInputs() 
1567              self._solver.setExtInputs(True, deepcopy(self._inputVarList),
 
1568                                      deepcopy(self._inputTimeList)) 
1569              self._solver.canContinue=True

1570  
 
1571  
 


1572 -    def __del__(self):


1573          genDB.unregister(self) 
1574          ODEsystem.__del__(self)

1575  
 
1576  
 
1577  
 
1578  # Register this Generator with the database
 
1579  
 
1580  symbolMapDict = {'abs': 'fabs', 'sign': 'signum', 'mod': 'fmod'} 
1581  # in future, provide appropriate mappings for libraries math,
 
1582  # random, etc. (for now it's left to FuncSpec)
 
1583  theGenSpecHelper.add(Radau_ODEsystem, symbolMapDict, 'c') 
1584
```

  


| Home | Trees | Indices | Help | PyDSTool |
| --- | --- | --- | --- | --- |

|  |  |
| --- | --- |
| Generated by Epydoc 3.0.1 on Fri May 4 15:24:26 2012 | http://epydoc.sourceforge.net |
